# Digital Health Technology Infrastructure Challenges to Support Health Equity in the United States: Scoping Review

**DOI:** 10.2196/70856

**Published:** 2025-09-15

**Authors:** Shongkour Roy, Stella T Lartey, Polina Durneva, Niharika Jha, Michael Arthur Ofori, Zebunnesa Zeba, Stella Dockery, Nichole Saulsberry Scarboro, Michelle Taylor, Ashish Joshi

**Affiliations:** 1 The University of Memphis School of Public Health, 326 FedEx Institute of Technology, Memphis, TN, 38111, United States, 1 9016565922; 2 Department of Public Health Sciences, College of Behavioral Science and Health Sciences, Clemson University, Clemson, SC, United States; 3 Department of Information Systems and Business Analytics, Loyola Marymount University, Los Angeles, CA, United States; 4 PH-IDEAS, The University of Memphis School of Public Health, Memphis, TN, United States; 5 Shelby County Health Department, Memphis, TN, United States

**Keywords:** digital health technology, health equity, health providers, technology and system, scoping review, PRISMA

## Abstract

**Background:**

Although digital health technology (DHT) is widely used in the United States at both hospital provider and individual levels, it is beset with several challenges that have contributed to inequities in the health service delivery. Previous studies have shown that health inequities observed may be amplified many times by DHT requirements.

**Objective:**

The objectives of this scoping review are aimed at synthesizing information on DHT inequities by exploring evidence that describes DHT infrastructure needs focused on promoting health equity in the United States and identifying key challenges both at the individual or patient level and at the health service provider’s level.

**Methods:**

We adapted Arksey and O’Malley’s scoping review guidelines in our review. PubMed, Web of Science, CINAHL, and PsycINFO were searched. We also conducted supplementary searches on Google Scholar. The inclusion criteria were peer-reviewed publications that broadly conceptualize or analyze DHT infrastructure from a health equity perspective and the challenges of DHT requirements between 2020 and 2024. We have screened the full text of articles using eligibility criteria such as studies that were included if they examined DHT infrastructure in the United States from a health equity perspective, discussed health disparities resulting from DHT interventions, or investigated the variables influencing health inequities connected to DHT. Two researchers (SR and ZZ) evaluated each citation individually at the title and abstract levels. The thematic approach and qualitative analysis determined this scoping review’s outcome.

**Results:**

Of the 628 research papers from the search, 27 were included in the analysis based on the inclusion criteria. In this review, we discussed factors such as older adult population, education, race, ethnicity, and socioeconomic status leading to health inequities in DHT. Patients and service providers face challenges related to health inequities in the use of DHT. The most common challenges for service providers were infrastructure and technical issues such as inadequate integration with existing workflows, user-unfriendly health information exchange interfaces, and lack of skilled staff, while for individuals or patients, this included limited broadband web-based access, cultural or linguistic appropriateness, and access to digital tools.

**Conclusions:**

The study identified that in the United States, DHT is an essential part of the delivery of health services; yet, it is saddled with key challenges leading to health inequities. Finding pragmatic solutions to these challenges can improve health equity in DHT.

## Introduction

### Background

Digital health technology (DHT) is being widely recommended as a revolutionary platform for seeking health care services, decreasing medical costs, and enhancing public health [1]. The use of DHT to enable individuals to access health and social care is rapidly increasing [2] due to changes in population structure, chronic diseases, and the emergence of infectious diseases (eg, COVID-19 [3]). Advancements in DHT have enabled health service providers to obtain required patient information, engage families, access medical data, and facilitate collaborative decision-making in the United States. Nevertheless, these achievements, substantial infrastructure-related challenges remain that prevent equitable access to DHTs. Insufficient broadband connectivity, limited access to digital devices, interoperability, and inadequate digital literacy disproportionately impact historically marginalized communities. Addressing these infrastructure gaps is vital for ensuring that all individuals can equitably obtain the benefits of DHT innovation. Previous research has demonstrated that the use of DHT can improve individuals’ comprehension of health-related information, elevate patient involvement in health care decision-making, enable patients to exert more authority over their health, and enhance the efficiency of health care delivery, especially for individuals with chronic illnesses [4-6]. The appropriate use of DHT has enhanced information accessibility and communication technology, making preventative services, treatment, and education to patients seamless [7]. It also facilitates illness tracking and monitoring, as well as empowers consumers to actively engage in health care services [8]. In addition to the patients and individual-level benefits, health services providers have alluded to the substantial positive impact of DHT enhancing health delivery, patients’ management, data-driven decision-making, and continuity of care. Although DHT enhanced patient management by assuming that patients have high digital literacy, English proficiency, and access to technology, marginalized populations with limited tech skills, including non-English speakers, older adults, and individuals with disabilities, may be left behind [9].

### Key Components of Digital Health

DHT encompasses the use of electronic health records (EHRs), electronic medical records (EMRs), telemedicine and telehealth, eHealth, and mobile health (mHealth), facilitated by smartphones, wearables, mobile apps, and monitoring devices [10-13]. While eHealth refers to EHR, patient administration systems, laboratory systems, and other records that cannot be stored within mHealth apps, mHealth is deemed appropriate for medical and health care that uses mobile devices, such as a cell phone or a tablet to support health care practices. With mHealth services, patients can log, store, and monitor their health records on their personal mobile devices [14]. Telehealth refers to both clinical and remote nonclinical services, including training and continued medical education for practitioners [15]. On the other hand, telemedicine solely refers to remote clinical services. EHR and EMR widely use DHT. EHR focuses on recording a patient’s medical history that is maintained electronically by multiple providers. Unlike EHR, which is designed to be interoperable, and that means the ability of different EHR systems to interact and transfer patient information effortlessly, EMR is not designed to be interoperable [16]. Implementing DHT has been found to enhance health care services, improve health outcomes, and reduce medical expenditures [16]. However, the rapid advancement of DHT while improving patient health outcomes and supporting health services provision has resulted in DHT inequities mostly at both the individual and health service provider levels [17].

### DHT Inequity in Patients and Providers

The health inequities resulting from DHT are widely recognized at both patient and provider levels globally [17]. DHT inequities refer to disparities in health status or the distribution of health care resources across different groups due to socioeconomic factors, such as people’s geographic origin, education, lifestyle, or work [18,19]. Despite the rapid adoption of DHT, it is likely that individuals who do not regularly use the web or mobile devices, or who face challenges in using them—such as the older adults, those in low-income communities, and people in remote areas with limited web-based access—will be overlooked [10]. This phenomenon signifies not only the inequities in wealth, education, age groups, and health status but also the capacity to access and use technology, which remains a persistent obstacle to the use of DHT services. Health service providers faced challenges during the COVID-19 pandemic in providing services to their clients due to a shortage of skilled staff, inadequate integration with existing workflows, and a user-unfriendly health information exchange (HIE) interface [10]. Several studies have shown that interventions targeting DHT at both patient and health service provider levels might worsen health care inequities [20]. DHT interventions have more efficacy among those who are already in a more advantageous position, hence resulting in inequities [20]. Marginalized populations [21] have more obstacles in obtaining and implementing DHT [10], resulting in exacerbated health inequities. Health equity is prioritized above health inequity due to the potential adverse social and economic outcomes associated with the latter [22].

### Global DHT and Health Equity

DHT holds transformative potential for achieving global health equity, but it requires addressing the root causes of inequity alongside technological innovation. Ghanem et al [23] performed a quick narrative analysis and emphasized that to use artificial intelligence (AI) successfully in public health without worsening existing disparities, health equity issues must be addressed. They identified several important problems pertaining to health equity in the implementation of AI, including shortcomings in AI epistemology, algorithmic bias, restricted accessibility of AI technologies, ethical and privacy dilemmas, unrepresentative training datasets, insufficient transparency and interpretability of AI models, and difficulties in scaling technical competencies in Canada. Likewise, Owoyemi et al [24] investigated digital solutions for community and primary health professionals in Africa, emphasizing major challenges to fair adoption. These included restricted network coverage, insufficient technical proficiency, irregular power supply, poor mobile phone adoption, and challenging application design [24]. Another research study conducted in Vietnam evaluated the use of DHTs across 5 hospitals and identified challenges such as the inadequate implementation of EMRs, lack of interoperability across systems, and insufficient infrastructure, all of which impede the equitable provision of health care services [25].

### Theoretical Model in DHT

In 2019, the National Institute on Minority Health and Health Disparities established a study framework for digital health equity [26]. This framework is organized around many essential areas, including the digital landscape and the health care system. The digital environment significantly impacts digital health equity at several levels: individual, interpersonal, communal, and societal. At the individual level, elements such as digital literacy, digital self-efficacy, access to technology, and attitudes toward technology use are essential. The interpersonal dynamics among patients, technology, and professionals are crucial. Community-level factors include health care infrastructure and dominant societal norms around technology use. At the social level, technological policy and data standards are crucial determinants of digital health equity. Currently, there is a lack of literature evaluation about the health disparities arising from the use of DHT at the patient, health care provider, and health and technology system levels in the United States. Consequently, we have conducted a scoping assessment of DHT within health care environments.

### Aim of Scoping Review

The aim of this scoping review will address is what the key challenges in DHT infrastructure are that impact efforts to achieve health equity in the United States? The specific aims include the following: (1) to determine factors within the DHT infrastructure for patients, providers, and technology and system levels that hinder equitable access to health care in the United States; (2) to analyze infrastructural limitations in DHTs that contribute to health disparities in the United States; and (3) to investigate the existing literature on what types of digital health infrastructure challenges are most reported in the literature as affecting health equity in the United States.

## Methods

### Overview

This scoping review aimed to synthesize information on DHT inequities by creating map review-level evidence that described DHT infrastructures focused on promoting health equity in the United States. Additionally, our goal is to identify key challenges, both at the individual or patient level and at the health service provider’s level. By identifying these challenges, we can develop potential programs or interventions to reduce health inequities in the United States and identify research gaps for future research and practice that will benefit policy makers and researchers.

Based on the defined aim, this scoping review included findings from a wide range of literature and identified areas where further research is needed. This review was conducted in accordance with the Arksey and O’Malley [27] framework. We outline the precise methodologies used in our scoping review as following stages:

### Stage 1: Identifying the Research Question

The study was based on the research question outlined in the objective. The PEO (Population, Exposure, and Outcome) technique helped us organize our study question and plan how to find the answers. The population is “individual or patients, and health service providers in the US,” the exposure is “DHT,” and the outcome is “health inequities/equities due to use of DHT.”

### Stage 2: Identifying Relevant Studies

We conducted the search process from January 2024 to February 2024, using the following search query: (“DHT”[Title/abstract] OR “mhealth”[Title/abstract] OR “telemedicine”[Title/abstract] OR “telehealth”[Title/abstract] OR “electronic health records”[Title/abstract] “electronic medical records”[Title/abstract] OR “infrastructure”[Title/abstract]OR “promoting”[Title/abstract] OR “challenges”[Title/abstract] OR “barrier”[Title/abstract] OR “providers competencies”[Title/abstract] OR “technical support staff”[Title/abstract] OR “affordable connectivity”[Title/abstract] OR “affordable device ”[Title/abstract] OR “DHT literacy”[Title/abstract] OR “reliable connectivity”[Title/abstract] OR “interchange information”[Title/abstract] OR“minimum”[Title/abstract]) AND (“health equity”[Title/abstract] OR “health inequity”[Title/abstract]) AND (“US”[Title/abstract] OR “United States”[Title/abstract]).

This time frame focuses on recent and policy-relevant data regarding DHT infrastructure (such as telehealth, remote monitoring, and EHR) challenges due to the initial year of the 2020 COVID-19 pandemic dramatic shift in the adoption and reliance on DHT. We also found that there was significant policy development in the United States related to digital health (such as Federal Communications Commission broadband initiatives, which are working to connect communities and supporting DHTs) in this time frame. We used a mix of terms that included search queries in English based on our eligibility criteria, including different combinations of the phrases to capture as many articles as possible. Before finalization of the search term in this review, 2 coauthors (SR and AJ) did a literature review. After completing the database searches, we reviewed references of all identified papers using backward snowballing and a list of publications that cited the included papers using forward snowballing. The snowballing was done using Google Scholar.

#### Criteria for Considering Studies for This Review

We included only peer-reviewed original papers, concept papers, review, and viewpoint in this study. This paper did not include any systematic review papers. However, papers listed in prior systematic reviews on DHT were thoroughly checked for inclusion criteria. We used quantitative, qualitative, and mixed data collection methods in this study.

#### Topic of Interest

We sought primarily to identify papers that focused on DHT infrastructure and health equity in the United States. For this review, we have considered DHT infrastructure and the domain of DHT as eHealth, mHealth, telehealth, telemedicine, EHR, and EMR. We have described DHT infrastructure as individual access and engagement, equity and ethics, data privacy and reliability, data storage, and management. Furthermore, we explored DHT and health equity in health service providers and patients or individual level.

#### Inclusion and Exclusion Criteria

We considered inclusion and exclusion criteria in this review. No restrictions were placed on study methodologies. Studies were included if they examined DHT infrastructure in the United States from a health equity perspective, discussed health disparities resulting from DHT interventions, or investigated the variables influencing health inequities connected to DHT. We focused on literature from the United States because studying DHT and health equity within the United States provides a focused, context-specific analysis that considers its unique health care system, persistent disparities, and leadership in digital health innovation. These insights can drive meaningful improvements in equitable health care delivery and inform global discussions on digital health accessibility. This review examines works published in the English language from 2020 to 2024. We excluded papers that reported on DHT interventions or programs that did not focus on health equity or inequity views, and the papers are needed to access the full text.

### Stage 3: Study Selection

After the initial search, the search results were uploaded into Zotero (which was developed by a nonprofit Corporation for Digital Scholarship) for screening of the papers. To determine eligibility for the scoping review, 2 review writers (SR and ZZ) screened a total of 628 paper titles and abstracts of the identified records separately. After that, we eliminated any redundant records. We then screened the papers for eligibility and inclusion using 2 steps. All publications that 1 or both review writers determined to be possibly relevant were retrieved into their full texts. Then the full text of selected papers was evaluated separately by 2 review writers (SR and NJ). Disagreements were settled by mutual discussion. The papers, after the first-level and second-level screening, were included for data abstraction, using a standardized tool. We included a PRISMA (Preferred Reporting Items for Systematic Reviews and Meta-Analyses; the PRISMA-ScR [Preferred Reporting Items for Systematic Reviews and Meta-Analyses extension for Scoping Reviews] checklist is provided in Checklist 1) flow diagram to illustrate our search results and the process of screening and selecting studies with exclusion and inclusion.

### Stage 4: Charting the Data

We developed a data extraction tool in Microsoft Excel. The authors devised a data-charting form encompassing the subsequent categories: objectives of published paper, manifestation of health inequities, influencing factors to health inequities, DHT infrastructure challenges for both patients and service providers level, and gaps created each challenge. The data-charting form was piloted by the research team among 5 papers and assessed to ascertain its comprehensiveness. The data for the remaining papers were retrieved by SR and NJ. The finished extraction tables were evaluated and analyzed by the 2 researchers (SR and NJ). The procedure was iterative, ensuring that the tables had all the relevant information necessary for developing the themes, as outlined in step 5.

### Stage 5: Collating, Summarizing, and Reporting the Results

The researchers used a data extraction form to systematically gather specific details about descriptive data. Data were collected from each paper to provide a comprehensive description of the following aspects: (1) the study’s characteristics, such as the author, publication year, objectives of research, area of practice, target population, and methods used; (2) the manifestation of health inequities through the implementation of DHT; (3) the infrastructure challenges to supporting health equity for both providers and individual or patients’ level to adopt DHT; and (4) which gap was created for each challenge. In this study, the researchers conducted a thematic analysis to get the findings of this scoping review.

## Results

**Figure 1.**
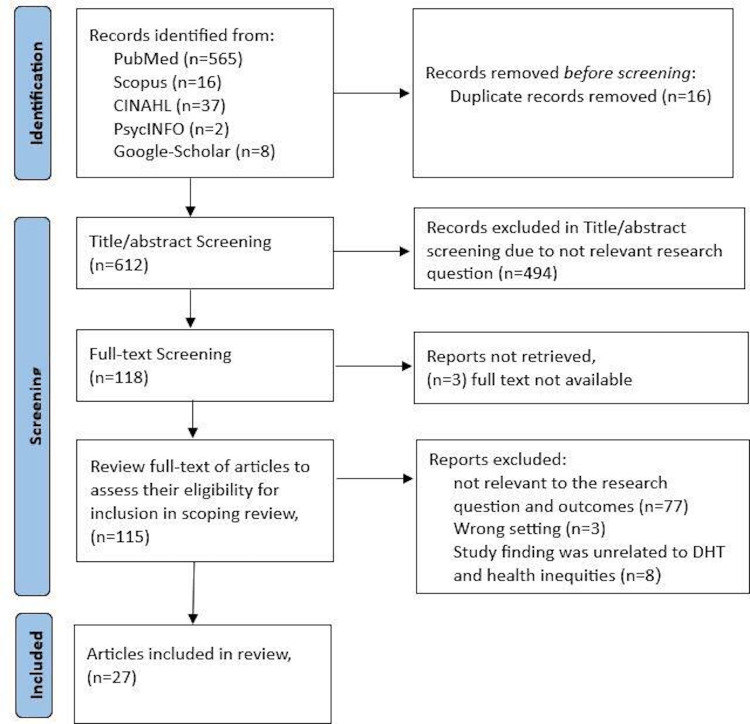
PRISMA (Preferred Reporting Items for Systematic Reviews and Meta-Analyses) diagram showing the flow of the search strategy and study selections. DHT: digital health technology.

The records of screened and identified studies are shown in the PRISMA diagram (Figure 1).

### Characteristics of Included Studies

A total of 27 studies, published between 2020 and 2024, satisfied the specified inclusion criteria discussed in the “Methods” section. Table 1 displays the categories used for charting and the corresponding information for the 27 studies. Tables2  3 illustrate the study methods and the different categories of participants or professionals. Fifteen studies used a quantitative research design. The study methodologies used included a variety of approaches, such as cross-sectional, retrospective, and cohort studies using interviews and questionnaires, as well as secondary data analysis. The outcomes of qualitative research using various interview methods, such as interviews, focus groups, key informative interviews, participatory interviews, and in-depth interviews, are described in 6 papers. One academic paper detailed the outcomes of DHT treatments using a combination of experimental methods, while another research used a combination of qualitative and quantitative methods (Table 2). Among the 27 studies included in this review, most publications focused on public health issues such as patients receiving obstetric care, family planning, safety and violence, general services, and cancer treatment (12 studies). Additionally, 4 studies examined health care practitioners as a professional group.

**Table 1.** Descriptive information of each study included in the review.

Study ID (first author, year)	Objectives	Manifestation of health inequities	Influencing factors of health inequities	DHT^a^ infrastructure challenges (service providers level)	DHT infrastructure challenges (individual and patients’ level)
Acholonu and Raphael (2022) [1]	To eliminate child health disparities in health care using the EHR^b^.	Provider’s racial bias to treat patients.	Racial bias.	EHR increases the burden of work for providers (patient portals may generate more work, confuse patients, and increase health disparities).	Portal uptake (patient portals enroll lower among racial and ethnic minority groups, older individuals, and those with lower education and health and computer literacy).
Antonio et al (2023) [4]	How health inequities reduce the use of telehealth by (1) logging into the patient portal, (2) signing telehealth consent on the patient portal, and (3) conducting a telehealth video test run.	Participants stated a lack of comfort with technology, access to technology, or skills required for a telehealth video visit, which manifest health inequalities.	Discomfort with telehealth video calls.	Providers’ endorsement and perceptions of who uses technology can influence patients’ technology adoption.	Technical problems and the unavailability of videos influenced the visit modality from video visit to phone.
Marshall et al (2022) [6]	Explore the perceptions of oncology health care professionals regarding how telehealth was used to provide care to patients with cancer undergoing active treatment during the COVID-19 pandemic.	Lack of web-based access for remote-area patients and an aging patient population had difficulties navigating the new technology.	Geographic, aging, lack of access to the internet, and racial or ethnic.	There was limited access to interpreters for the telehealth platform.	Patients did not have smartphones or access to computers and the internet. Patients who were non–English-speaking often had difficulty communicating via telehealth.
Nelson et al (2022) [12]	Digital health care literacy.	Despite the potential for DHT care to improve quality of life and clinical outcomes, many individuals may not have the skills to engage with and benefit from it.	Education, income, and skills.	—^c^	Participants who did not have digital tool access had lower scores than those who did. Specifically, participants who did not own a smartphone or a laptop computer had lower digital literacy scores.
Verma et al (2022) [13]	Underrepresented individuals face multilevel DHT disparities that potentially diminish the benefits of these digital advances.	Patients with high school education or less were less likely to use telehealth than those with a bachelor’s degree or higher.	Education, age, income, and ethnicity	—	People whose incomes are below the federal poverty threshold, Hispanic, and Asian patients were more likely to use telehealth than non-Hispanic White and Black patients.
Blount et al (2023) [10]	To assess the adoption and use of DHTs by primary care clinicians in southeastern states and identify individual-level and practice-level barriers and facilitators to DHT implementation.	Rapid adoption of DHTs has contributed to inequitable use and benefit.	Lack of monitoring inequitable use and benefit.	Time and cost and limited workflow integration were identified as barriers. Incomplete and difficult-to-use HIE^d^ interfaces for providers.	Internet or broadband access and poor connectivity for patients.
Pronk et al (2021) [22]	Healthy People 2030 describes a vision and offers benchmarks that can be used to track progress toward the goal of all people in the United States achieving their full potential for health and well-being across the life span.	Evidence-based interventions and policies are important to reduce health inequity.	Born, living place, and age.	—	Good health and well-being flourish across geographic, demographic, and social sectors; fostering healthy equitable communities guides public and private decision-making; and everyone can make choices that lead to healthy lifestyle.
Udegbe et al (2023) [28]	Telemedicine has existed for decades as a means of providing care to those in rural areas.	Although the benefits of telemedicine are important to many patients receiving care, there are large barriers to achieving equity for certain communities. The most apparent is the “digital divide” that results from lack of necessary technology in certain groups of people or specific communities, such as rural areas.	This includes the lack of broadband web-based access, technology literacy, and availability of appropriate devices for virtual visits.	—	Phone telehealth visits were covered by Medicaid at a lower rate, although for some communities, landlines are more reliable than using cellular or web-based data for video visits.
Kim and Backonja (2024) [29]	How do community-based organizations develop intervention for DHT and health equity?	Access and use of telehealth and other DHT solutions, health care usage, health/clinical/psychosocial outcomes, caregiver health, and wellness.	Web-based access and digital literacy.	—	Web-based access challenges the need for access to devices and digital literacy.
Lyles et al (2022) [30]	Outlines the pillars of a health equity framework from the Institute for Health Care Improvement, overlaying a concrete example of telemedicine equity.	Racial or ethnic, linguistic, and socioeconomic groups in the United States faced health inequity by telemedicine.	Telemedicine.	Many health care systems have reported on patients’ self-reported race or ethnicity and preferred language within EHR (this needs to be a universal standard for every health care system in the country).	Broadband access at the census tract level is associated with uptake of DHT.
Chen et al (2022) [31]	To better understand the association between PHS^e^ partnerships, telehealth post discharge, and racial and ethnic disparities in health care.	Black and Hispanic Medicaid FFS^f^ beneficiaries were less likely to be treated in hospitals that provided telehealth postdischarge services than White patients.	PHS integration and care coordination through health information systems.	—	DHTs are not designed by reflecting patients’ cultural background and preferences.
Dixit et al (2022) [32]	Multilevel barriers to telehealth and proposes steps to address barriers and inequities in telehealth.	Telehealth digital systems are complex and not designed for accessibility for older patients and for patients who may have limited digital literacy.	Lack of facilities, such as patient health systems and telehealth portals, is not designed for individuals with low education.	There is a lack of trained providers and a lack of optimized workflow.	Patients have inexperience with telehealth, access to devices, and broadband.
Chunara et al (2021) [33]	Black patients faced disparity in accessing telemedicine.	There are disparities for Black patients who are accessing telemedicine. Median income and decreased mean household size of a zip code were also significantly related to telemedicine use.	Race, income, and geography.	Culturally and structurally appropriate telemedicine tools.	Access to telemedicine.
Lyles et al (2021) [34]	Digital access and skills are foundational social determinants of health.	Lack of a specific focus on equity risks building digital solutions that improve the health outcomes only for selected, advantaged individuals, without improving overall outcomes or decreasing entrenched health disparities.	Patient’s race and ethnicity, socioeconomic status, language, digital literacy, and health literacy.	Building and testing tools in the populations that need and can benefit from them offer the best opportunity to ensure that the health care digital revolution improves health equity.	Access to digital infrastructure, including device ownership and availability of broadband, still lags in the United States compared with other developed countries.
Ko et al (2023) [35]	Lack of access (and awareness of access) is likely a primary driver of disparities in rural telehealth use.	Rural and low-income adults were less likely to report telehealth access than nonrural and non–low-income counterparts.	Geography and income level.	—	Access to telehealth among rural and low-income.
Koenig et al (2023) [36]	The role of telehealth in mitigating inequities in abortion access.	Telehealth can play a key role in accessing an otherwise unobtainable or delayed abortion, especially for marginalized patient populations.	Travel distance, time, and costs, based on geospatial location, socioeconomic status, and local abortion policy.	—	While states that ban abortion are unlikely to support telehealth for abortion, there are 6 states that permit abortion but restrict telehealth abortion care.
D'Amico et al (2023) [37]	Sociodemographic differences in primary care via telehealth compared with in-person office visits before and during the COVID-19 pandemic.	Encounters with the following patients were less likely to be via telehealth than via in-person office visits.	Age, self-identified race, ethnicity, zip code, and insurance type.	—	Compared with patients who identified as White, encounters during 2020 with patients of the following racial or ethnic groups were significantly less likely to be via telehealth. Medicare patients were less likely to use telehealth.
Bhagavathula and Aldhaleei (2024) [38]	Identifying trends and disparities can inform efforts to promote greater awareness, accessibility, and inclusivity in telehealth services, and addressing disparities is critical to ensuring equitable access to health care through telehealth.	Significant disparities remain among underserved populations, highlighting the need for targeted efforts to increase access and reduce barriers.	Low coverage of telehealth.	—	Lack of digital access, technological literacy, awareness, and cultural or linguistic appropriateness.
Haimovich et al (2024) [39]	Provide characterization of electronic behavioral alerts using EHR data across a large, regional health care system.	Black and Hispanic Medicare FFS beneficiaries were less likely to be treated in hospitals that provided telehealth postdischarge services than White patients.	Rural and low-income adults were less likely to report telehealth access than nonrural and non–low-income counterparts.	Geography and income level.	—
Berwer et al (2020) [40]	How technological advances can lead to unintended consequences such as perpetuating health and health care disparities for underresourced populations.	Racial and ethnic minorities.	Disadvantaged groups, including racial and ethnic minorities.	—	Peer- and Technology-supported self-management training.
Gaziel-Yablowitz et al (2021) [41]	Local hospital characteristics and regional policy features that were associated with adoption and implementation of telehealth. Telehealth policies should be national rather than at the state level to promote the use and equity.	Hospitals that were nonprofit, major teaching, affiliated with a health system, and micropolitan were more likely to adopt DHTs.	Hospital investment in telehealth was disproportionally distributed between several telehealth capabilities.	Reimbursement policies.	—
Erikson et al (2022) [42]	Association of payment policies and financing with telehealth usage.	Due to the fiscal constraints facing the financial sustainability of telehealth, the relationship between telehealth usage and payment parity may be highly relevant.	Financial sustainability, payment parity policies, and continued investments in broadband availability.	Low investment in broadband availability in rural and underserved communities.	Supportive payment policy.
Franciosi et al (2021) [43]	Comparing clinic visit and hospital visit patients’ specialty-specific (pediatric surgical, adult, and nonsurgical) changes in patient demographics, including a younger population, fewer non–English-speaking patients, and a relative preservation of minority, Medicaid, and Medicare patients.	Restriction on insurance coverage for telephone visits.	Lack of insurance coverage.	Nonsurgical specialties largely benefited from telephone visits, whereas surgical specialties did not.	Non–English-speaking patients have more challenges in health inequity.
Heintzman et al (2023) [44]	Non–US-born, US-born, and patients without a country of birth record in EHR are more often uninsured for health services such as in diabetes, hypertension, and hyperlipidemia.	Country of birth or nativity information is crucial to understanding health equity.	Lack of unified EHR.	Community health centers are not collecting birth information data by EHR for all Latino populations.	—
Levine et al (2022) [45]	To assess characteristics associated with telehealth adoption.	Invested in telehealth consultation services and stroke care. Nonprofit hospitals, affiliated hospitals, major teaching hospitals, and hospitals located in micropolitan areas (those with 10,000-50,000 people) were more likely to adopt telehealth.	Hospitals that lacked electronic clinical documentation, were unaffiliated with a hospital system, or were investor-owned had lower odds of adopting telehealth.	—	Patients did not provide telehealth services due to lack of telehealth adoption in rural areas than in micropolitan areas.
Sbruzzi et al (2023) [46]	Access to ICTs^g^ in pediatrics care.	People may have access to the internet, but that does not indicate that they have the necessary skills to use it and to benefit from it.	Necessary skills to use technologies.	—	Ensuring connectivity is not enough to guarantee access to ICTs. The use and benefits obtained also depend on other factors, such as socioeconomic level, education, and personal motivations described as determinants for interaction with web-based activities.
Shin et al (2025) [47]	To characterize their global learning experience with respect to community context, the learning and implementation process, implementation science considerations, and health equity.	Community engagement and ownership are core to the implementation process and goal of health equity.	Rural communities with limited resources and their own health equity challenges, such as long distances for residents to access grocery stores and health facilities.	Organizational culture and infrastructure are important for eliminating health disparity.	Trust building in the community can help reduce disparity.

a DHT: digital health technology.

b EHR: electronic health record.

c Not available.

d HIE: health information exchange.

e PHS: public health systems.

f FFS: fee-for-service.

g ICT: information and communication technology.

**Table 2.** Study designs of included studies.

Study methods	Number of studies [references]
Face-to-face interview In-depth interview Observation	1 [4]
Focus group discussion Face-to-face interview	1 [10]
Focus group discussion Literature review In-depth interview Key informant interviews Participatory research	7 [12,22,29,30,32,34,46]
Interview, retrospective analysis, cross-sectional, and cohort	15 [12,17,28,31,33,36,37,39,41-45,47,48]
Review and viewpoint	3 [1,12,46]

**Table 3.** Type of participants in included studies.

Participants	Number of studies [references]
Patients and individuals	23 [4,12,13,22,28-37,39,41-47,49]
Service providers	4 [1,10,12,17]

### Aggregation of Key Statistics of Included Studies

In this review, 5 studies specifically addressed the challenges associated with web-based and broadband access in the context of DHTs’ health inequities [10,17,28-30]. Limited broadband availability and unreliable web-based infrastructure were frequently cited as significant barriers, particularly in rural and underserved communities, where these challenges exacerbate existing disparities in access to DHT services. Among the most reported influencing factors across the studies were low patient portal adoption rates [1,4,10,31,32], lack of access to digital devices or reliable web-based connectivity [12,33,34], and limited technological literacy [28,29,33,34]. These interrelated barriers contribute to persistent inequities by limiting the ability of certain populations—especially older adults, individuals with low socioeconomic status, and racial or ethnic minorities—to fully engage with and benefit from DHTs.

### Thematic Analysis

We identified 4 themes related to DHT focus on promoting health equity and their key challenges for both individuals or patients and health service providers' level.

#### Theme 1: Factors Leading to Health Inequities at DHT

**Figure 2.**
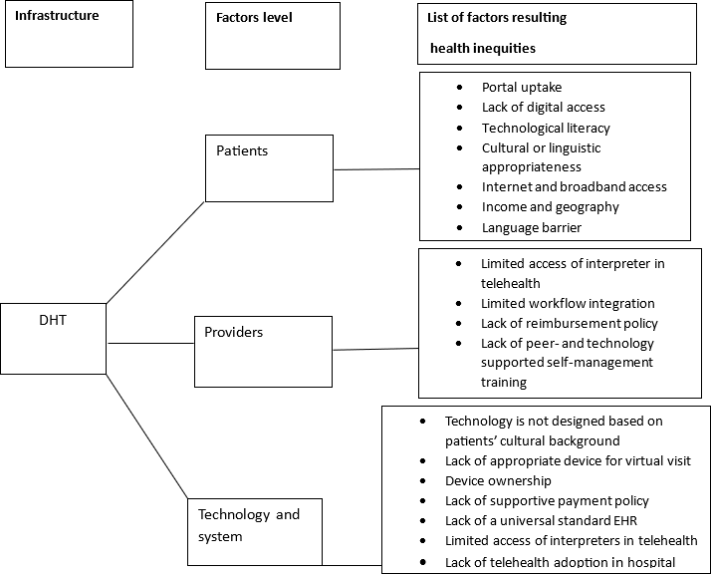
Diagram of influencing factors resulting in health inequities in the digital health technology infrastructure. DHT: digital health technology; EHR: electronic health record.

Most papers (91%) investigated the factors (Figure 2) that lead to inequities in health in connection to DHT. Five studies have shown that age is a major factor, regardless of whether patients use DHT. This was particularly evident among the older adult population [50]. The role of race or ethnicity in contributing to health inequities has been well recognized [35,51,52]. However, the potential health advantages of eHealth or mHealth programs have not been effectively used within Black communities. The relationship between race and ethnicity and health inequities has been acknowledged for a considerable period [31]. The use of eHealth or mHealth systems, which are recognized for their advantages, has not been generally adopted by Black communities [52]. In rural areas with many health care barriers, the dissemination of health information becomes more difficult, which has the potential to worsen the problem rather than reduce inequality [36]. Moreover, socioeconomic status [37], including factors such as level of education and possession of assets, had a substantial influence [53] in health inequities. Multiple studies have shown inequities in the buying and use behaviors of DHT devices across different demographic segments, as determined by their income and education levels [7]. In addition, several studies have particularly investigated the impact of wealth [54] and educational achievement [8,33] on DHT separately.

#### Theme 2: Health Inequities Resulted by DHT at Patient’s Level

This theme focused on using DHT to analyze inequities in health at the patient and individual levels. Amidst the COVID-19 pandemic, Chunara [33] executed cohort research inside a prominent health care system in New York City, examining the relationship between telemedicine and health care inequities. Black people exhibited less likelihood of using telemedicine for health care services than White patients. However, there has been a growing trend among Black patients, particularly among younger individuals and females, to seek telemedicine for urgent medical needs. Bhagavathula and Aldhaleei [38] performed research using equity-focused methodologies to examine the growing discrepancy in health inequities, despite the development of telehealth services throughout the pandemic. This research identified challenges to telehealth accessibility among disadvantaged communities, such as limited web-based access, technical proficiency, awareness, and cultural and language appropriateness. Another study [48] investigated the premise that there are systematic inequities in the use of DHT services among individuals who reside at or below 200% of the federal poverty threshold. This study pointed out that telehealth use was more prevalent among patients of Hispanic and Asian descent, as well as those with incomes below the federal poverty level, than among non-Hispanic White and Black patients. The paper’s findings indicate that education, age, income, and ethnicity play crucial roles in leveraging DHT to mitigate health inequities. Udegbe et al [28] investigated the challenges to establishing fairness in telemedicine for obstetrics in rural regions. This research highlighted that although telemedicine offers significant advantages to many people seeking medical treatment, there are major challenges in ensuring equal access for certain groups. One prominent issue is the “digital divide” resulting from a shortage of essential technology among some demographics or certain regions, such as those residing in rural areas. Haimovich et al [39] conducted a study that described the features and functions of electronic behavioral alerts in emergency departments. The study used EHR data from a large, regional health care system. Hospitals offering telemedicine postdischarge services had a lower likelihood of treating Black and Hispanic Medicare enrollees in comparison with their White counterparts. Brewer et al [40] examined the potential negative outcomes that might arise from technological progress, including the perpetuation of health and health care inequities for disadvantaged groups. Additionally, Blount et al [10] discussed that web-based or broadband access and poor connectivity for the patients raised health inequities when they are seeking services for their health.

#### Theme 3: Health Inequities Resulted by DHT at Provider Level

This theme focused on the DHT applications of health service providers, which resulted in health inequities due to various systematic barriers and challenges. Research using qualitative analysis and opinion-based methodologies has highlighted the disparities arising from DHT implementation among health service providers. Acholonu and Raphael [1] reviewed the existing research on the impact of the EHR in promoting fairness and reducing disparities in health care, revealing that racial biases among providers influence treatment decisions. Specifically, Black children are less inclined to provide opioid medication for pain management when these patients appear with appendicitis or a long-bone fracture in the emergency department. Similarly, Blount et al [10] examined the acceptance and use of DHT by primary care providers in southern states. This study found key barriers and factors such as limited provider time, high costs, and poor integration with existing workflows that promote health inequities by restricting access to equitable care. Additionally, Antonio et al [4] reported to his publication that the endorsements and views of doctors may have an impact on the adoption of technology by patients. Health service providers identified that health inequities were more prevalent during video consultations compared with phone calls, resulting in a higher occurrence of technical difficulties for patients. Moreover, inadequate and user-unfriendly HIE interfaces at the providers’ level contribute to health inequities. Dixit et al [32] examined the multilevel challenges in the implementation of telehealth, emphasizing that the shortage of skilled staff and the absence of optimized workflows further contribute to disparities in access and quality of care. Collectively, these findings underscore the critical role of DHT at the provider level in perpetuating health inequities, necessitating targeted interventions to enhance accessibility, usability, and fairness in digital health care systems.

#### Theme 4: Discriminatory Adoption of DHTs Increased Inequities in the Technology and System Level

Integrating DHT into the design and development of specific DHTs is crucial at both patient and individual levels. Gaziel-Yablowitz et al [41] conducted a study in the United States on the adoption of DHT in hospitals, revealing that 73% (out of 2923 hospitals) had at least 1 DHT capability. More than 50% of these institutions allocated resources to DHT consulting services and stroke treatment [13]. Hospitals categorized as nonprofit, associated, major teaching, or situated in micropolitan regions (with a population of 10,000‐50,000) had a higher propensity to adopt DHT. Conversely, hospitals that did not have electronic clinical documentation, were not associated with a hospital system, or were controlled by investors had a reduced likelihood of adopting DHT. Therefore, the logistics of unequal adoption of DHT in the hospital system increased health inequities by exacerbating disparities in access, quality, and efficiency of care. There was no relationship between any of the statewide policies and the adoption of DHT. Erikson et al [42] discussed the impact of payment systems and financing on the use of DHT in community health clinics. The researchers found that the financial stability of DHT in community health centers is closely related to the correlation between telehealth use and payment parity. In addition, they addressed the topic of sustaining investments in broadband infrastructure in rural and underserved regions.

#### Theme 5: Designing Intervention of DHT Focused on Promoting Health Equity

This theme discussed studies that investigated the impact of DHT interventions in addressing health inequities. These interventions included activities such as accessing the patient portal, providing telehealth consent via the patient portal, and completing a trial telehealth video session [4]. The intervention research assessed the factors contributing to participants’ web-based connection issues during telehealth sessions and applied telehealth video run-test solutions. This research revealed that those who expressed discomfort and limited availability of technology saw more gaps in health. Individuals in the intervention group reported a heightened comprehension of telehealth, although they had more difficulty in learning the video visit software than individuals in the nonintervention group. Participants who are not included in the intervention need additional technological help and the necessary abilities for DHT. The intervention proposes that patients may need better access to web-based technologies and assistance from human intermediaries to effectively use DHT. In their paper, Kim and Backonja [29] examined how community-based organizations devised interventions to address DHT and health equity. They emphasized the significance of web-based access challenges, the necessity of device accessibility, and the importance of digital literacy for community-based organizations involved in interventions aimed at achieving DHT equity.

## Discussion

### Principal Findings

This study used scoping review methods to provide a comprehensive overview of the key challenges within DHTs infrastructure that affect efforts to achieve health equity in the United States. Our findings highlight that health inequities related to DHTs have become increasingly prominent in recent years. We identified several critical infrastructure factors contributing to these disparities. One major barrier is limited web-based and broadband access, especially in rural and underserved communities, where inadequate connectivity worsens existing challenges in accessing digital health services. In addition, we found that other interconnected barriers—such as lack of access to digital devices, unreliable web-based service, and low levels of technological literacy—further limit the ability of certain populations to benefit from DHTs. These challenges disproportionately affect older adults, individuals with low socioeconomic status, and racial and ethnic minorities, thereby reinforcing persistent health inequities.

This scoping review maps and describes the health inequities present for both health service providers and patients in the use of DHT. Publications from 2020 to 2024 in the United States used in this review showed an increased use of DHT during this time frame, as well as pervading health inequities. Our analysis of these studies identified several key indicators that influence health inequities at both the patient and individual levels. The indicators include age (particularly older people), race or ethnicity, income, health literacy, geographical location, broadband access, and poor connectivity. Age was identified as one of the major factors, particularly among the older adult population, regardless of whether they used DHT. The role of race and ethnicity in health inequities was well recognized. Despite the potential benefits of eHealth and mHealth programs, these technologies have not been widely adopted within Black communities, limiting their health efficacy. In rural areas, where health care barriers are prominent, the dissemination of health information through DHT is more difficult, potentially exacerbating inequities instead of alleviating them.

Our study shows that patients who have less education and lack knowledge in health and digital literacy might face challenges while trying to register for the patient portal. Nevertheless, the patients who have older adult population across various age groups exhibit a greater need for long-term access to health care services [55] and also face difficulties in registering for the patient portal, highlighting an inequity in the use of DHT across various age groups. These disparities are further amplified by age-related barriers, such as declining vision, hearing, and cognitive function, which are important to the use of DHT. These findings are consistent with previously published studies in that older individuals are more vulnerable to experiencing unequal opportunities to access and the use of DHT resources due to their limited understanding of digital literacy [56]. Individuals in the age range of 18-44 years who had obtained a bachelor’s degree were shown to have a greater likelihood of using telehealth services [57]. Health literacy is a necessary condition for effectively addressing a health issue using the DHT system [58]. Individuals must acquire a deeper comprehension of DHT, including mHealth apps, and the process of accessing health information over the internet. Our study revealed that a lack of education contributes to a lower level of health literacy, which hinders the community’s capacity to get and use health-related information. This finding is compatible with that of authors Williams and Shang [48], who worked on telehealth usage among low-income racial groups and found that telehealth exacerbates health inequities due to the digital divide. We have found that income-based health inequities are widespread in society, with people of better income having more access to DHT resources. Several studies specifically investigated how wealth and educational attainment separately impact DHT adoption, showing a clear link between these factors and inequities in DHT access and use. Additionally, Williams et al [59] referred to people who belong to the federal poverty level as the fundamental factor of health inequities, and patients who are employed full-time were more likely to use telehealth.

EHR is one of the major components of DHTs. Our study emphasized that the demographic information of patients from EHR data at the time of treatment decisions among health providers can influence racial biases, contributing to disparities. We found that health providers have time constraints to use DHT, and they could not integrate workflows perfectly due to lack of training, which factors limit access to equitable care and perpetuate health inequities. The lack of digital device access for limited-income individuals is one of the major factors limiting access to equitable care. To address this issue, this study pointed out that the United States implemented the “Affordable Care Act” to address the issue of health care access for low-income individuals. This legislation aimed to improve the availability and accessibility of health care information and resources for those who were uninsured and had low incomes [43,60], and this finding agrees with our results. Furthermore, our studies revealed that the lack of web-based or broadband access, as well as inadequate connections for patients, contributes to health inequities. Erikson et al [42] identified similar findings that a notable inequity in the availability of broadband and digital services has a direct effect on rural and socioeconomically disadvantaged areas. However, we found that regardless of access to the internet, individuals may not have the necessary skills to use it and to benefit from it, which may result in health inequities [44].

At the provider level, we identified health inequities for the use of DHT: the shortage of skilled staff, providers’ time, inadequate integration with existing workflows, and user-unfriendly HIE interface. The shortage of skilled staff in DHT is particularly concerning, as the lack of trained staff can prevent the efficient use of digital tools in delivering health services [45]. To achieve DHT equity, service providers must possess the ability to adjust their workflow in response to the use of new DHT [47,48]. To maximize the benefits of using digital tools [46], it is crucial to rethink workflows while enhancing current digital infrastructure, since these technologies typically facilitate continuous work, such as in-person visits or service supply. This finding was consonant with previous published study [61]. When effectively included, digitally empowered processes may optimize the efficiency of HIE, increase the quality and safety of services, and promote care coordination in health systems. In addition, health inequities may occur when there is a lack of adequate skills or support for creating processes, especially in settings that focus on disadvantaged groups that may need extra time and assistance to adopt digital platforms. The most related scoping reviews to this work are the DHTs and inequalities by Badr et al [62]. They have focused on the fact that DHTs can reinforce inequalities within vulnerable populations. However, they have limited focus on provider and technology and system level. Our study provided details on the contribution of DHTs’ health inequities on provider and technology and system level.

The integration and adoption of DHT at both the systemic and individual levels significantly impact health equity. At the provider level, barriers such as the shortage of skilled staff, time constraints, and inadequate workflows limit DHT’s potential to reduce health disparities. Additionally, systemic issues such as hospital categorization and payment systems further exacerbate inequities. Interventions designed to bridge technological gaps, improve digital literacy, and enhance access to DHT are essential to promoting health equity, especially for underserved populations. Addressing these barriers and designing inclusive DHT systems will be key to ensuring equitable health care delivery in the digital age.

### Research Gaps

This study has identified research gaps in existing literature. Most studies used a combination of quantitative and qualitative methodologies, known as mixed methods, to investigate how various factors at the patient, individual, and provider levels contribute to health inequities in the field of DHT. The factors included in this list are the aging population, income levels, health literacy, organizational culture [47], availability of internet access and poor connection, insufficient number of workers with digital skills, inadequate integration of workflows, and HIE systems that are difficult for patients to navigate. Subsequent research can use health informatics methodologies, such as human-centered design and AI-driven data analysis, to investigate the impact of additional indicators on health inequities. This can provide insights into the interrelationships between indicators in terms of occurrence and explore the causal pathways connecting these indicators. Additionally, these approaches are particularly valuable in bridging the gap between DHTs and health equity.

Our study comprehensively identified most factors affecting health equity at the individual, patient, and provider levels. Nevertheless, we found that objective factors, such as individual perspective on DHT, also have an impact on health equity. Our literature review revealed that younger age and education had a significant impact on DHT and health literacy. However, we did not find any evidence of moderating effects with other characteristics in relation to measuring DHT and health inequities. As a result, future studies may include subjective factors at both individual and provider levels and potential moderating influences when assessing DHT and health inequities. Additionally, our review highlights a lack of research on how clinicians use DHT interventions to support rural and disadvantaged populations. Subsequent studies need to include DHT interventions aimed at mitigating health inequities by offering tailored services to underprivileged and remote regions.

### Strengths and Limitations

The study has comprehensive mapping of health inequities due to use of DHT. We have identified key determinants by analyzing factors such as age, race or ethnicity, income, health literacy, and broadband access, which provided a thorough understanding of the multifaceted contributors to digital health inequities. This study has several limitations. One limitation of this study is the broad scope of the review question, which encompasses heterogeneous elements such as DHT infrastructures contributing to inequities and individual characteristics linked to inequitable access to DHT. This wide range of factors may have made it challenging to maintain a focused analysis and draw specific, actionable conclusions.

We searched the literature only in the 4 databases of PubMed, Scopus, CINAHL, and PsycINFO, which may limit the results from related literature that are not retrieved from other databases. The related search of DHT and health inequities results in many of them being qualitative in nature; therefore, we could not measure meta-analysis. We have excluded papers other than US settings, which might have led to the exclusion of relevant literature that could possibly have been used to widen or further support perspectives presented in our results. However, existing research does not examine how human-centered design approach helps implementers understand and navigate digital health tools in the implementation process more closely to reduce health inequity. Therefore, the identified opportunity in the literature highlights a need to conduct further research in this area. We have not focused the international literature on digital health inequities in this review.

### Conclusions

The scoping review analyzed the current literature addressing DHT and health inequities in the United States. Our findings highlight the inequities in health that arise from the use of DHT, influenced by multiple factors at both the patient and individual levels and the provider level. In response, we have identified interventions for DHT that specifically aim to promote equity in health. To address these inequities, relevant stakeholders such as policy makers, government and nongovernment entities, medical institutions, the DHT analyst, health care providers, and individual and patient health service receivers all need to initiate relevant actions to reduce inequities.

## Supplementary material

10.2196/70856 Checklist 1 PRISMA-ScR (Preferred Reporting Items for Systematic Reviews and Meta-Analyses extension for Scoping Reviews) checklist.

## Abbreviations

AI: artificial intelligence
DHT: digital health technology
EHR: electronic health record
EMR: electronic medical record
HIE: health information exchange
mHealth: mobile health
PEO: Population, Exposure, and Outcome
PRISMA: Preferred Reporting Items for Systematic Reviews and Meta-Analyses
PRISMA-ScR: Preferred Reporting Items for Systematic Reviews and Meta-Analyses extension for Scoping Reviews
